# Quantitative probability estimation of light-induced inactivation of SARS-CoV-2

**DOI:** 10.1038/s41598-024-54006-y

**Published:** 2024-02-09

**Authors:** Jaime Quintana, Irene Alda, Javier Alda

**Affiliations:** 1 Applied Optics Complutense Group, University Complutense of Madrid, Av. Arcos de Jalón, 118, 28037 Madrid, Spain; 2 School of Science and Technology, IE University, Paseo de la Castellana, 259E, 28029 Madrid, Spain

**Keywords:** Ultraviolet light, Optical disinfection, Light-virus interaction, Biophysics, Optics and photonics

## Abstract

During the COVID pandemic caused by the SARS-CoV-2 virus, studies have shown the efficiency of deactivating this virus via ultraviolet light. The damage mechanism is well understood: UV light disturbs the integrity of the RNA chain at those locations where specific nucleotide neighbors occur. In this contribution, we present a model to address certain gaps in the description of the interaction between UV photons and the RNA sequence for virus inactivation. We begin by exploiting the available information on the pathogen’s morphology, physical, and genomic characteristics, enabling us to estimate the average number of UV photons required to photochemically damage the virus’s RNA. To generalize our results, we have numerically generated random RNA sequences and checked that the distribution of pairs of nucleotides susceptible of damage for the SARS-CoV-2 is within the expected values for a random-generated RNA chain. After determining the average number of photons reaching the RNA for a preset level of fluence (or photon density), we applied the binomial probability distribution to evaluate the damage of nucleotide pairs in the RNA chain due to UV radiation. Our results describe this interaction in terms of the probability of damaging a single pair of nucleotides, and the number of available photons. The cumulative probability exhibits a steep sigmoidal shape, implying that a relatively small change in the number of affected pairs may trigger the inactivation of the virus. Our light-RNA interaction model quantitatively describes how the fraction of affected pairs of nucleotides in the RNA sequence depends on the probability of damaging a single pair and the number of photons impinging on it. A better understanding of the underlying inactivation mechanism would help in the design of optimum experiments and UV sanitization methods. Although this paper focuses on SARS-CoV-2, these results can be adapted for any other type of pathogen susceptible of UV damage.

## Introduction

The use of optical radiation at the ultraviolet C band (UV-C) is considered a reliable method to sanitize and disinfect environments and objects that could transmit diseases caused by virus, bacteria, or some other pathogens^1–7^. The bio-photochemical mechanism that causes virus inactivation appears to be linked to the photo-induced entanglement of RNA adjacent units when a photon, with adequate energy, interacts with the RNA strand^8–10^. The photon energy, or wavelength, required to induce pathogen photochemical inactivation is available in the scientific literature for a collection of different pathogens since the beginning of the 20th. century^4,11–14^. In terms of deposited energy, or dose, this parameter is typically defined as the fluence for a given level of inactivation (e.g., D63, D90, D99, etc).

In the last years, there has been a rise in the number of available studies regarding different aspects of SARS-CoV-2 virus—morphology, physical, and biological characteristics—due to the COVID-19 worldwide public health emergency. In this publication, we focus on further understanding how UV light causes photochemical damage in viruses. We go beyond the calculation of fluence, and estimate the number of photons interacting with the RNA chain. In “Geometric and genomic parameters for photochemical inactivation” section, we describe the current state-of-the-art regarding the geometry and genomic parameters of interest for our estimation. This approach allows us to calculate the average number of photons causing inactivation. In this analysis, we discuss and approximate the cross section of the virus and the internal RNA structures. Following the published results on photochemical damage of the RNA chain^10,14^, we evaluate the number of sites where the UV light may cause severe disruption of adjacent pyrimidine nucleotides. Then, in “Inactivation model” section, we test the binomial distribution to analyze the link between affected locations in the RNA chain and the inactivation of the pathogen. In this section, we have also incorporated a derivation that includes the well-established exponential surviving decay formula when this equation is given in terms of photon flux and photon numbers. Finally, we present our main conclusions in “Conclusions section”.

## Geometric and genomic parameters for photochemical inactivation

In this section, we use the available information about the shape and size of the SAR-CoV-2 pathogen to analyze how these geometric and physical parameters affect its interaction with UV radiation. For our estimation, we have also considered the virus’ capsule, where the RNA chains are organized in bundles. We employed the publicly available genomic sequence of SARS-CoV-2 to evaluate the number of occurrences of adjacent nucleotides susceptible to UV-C radiation damage. We have conducted the same analysis for a random RNA sequence—of similar length of the SARS-CoV-2—to check that, in terms of RNA neighbor pairs, the COVID-19 pathogen could be considered similar to a random sequence, enabling the expansion of this analysis to other pathogens.

### Geometrical characterization

**Figure 1:**
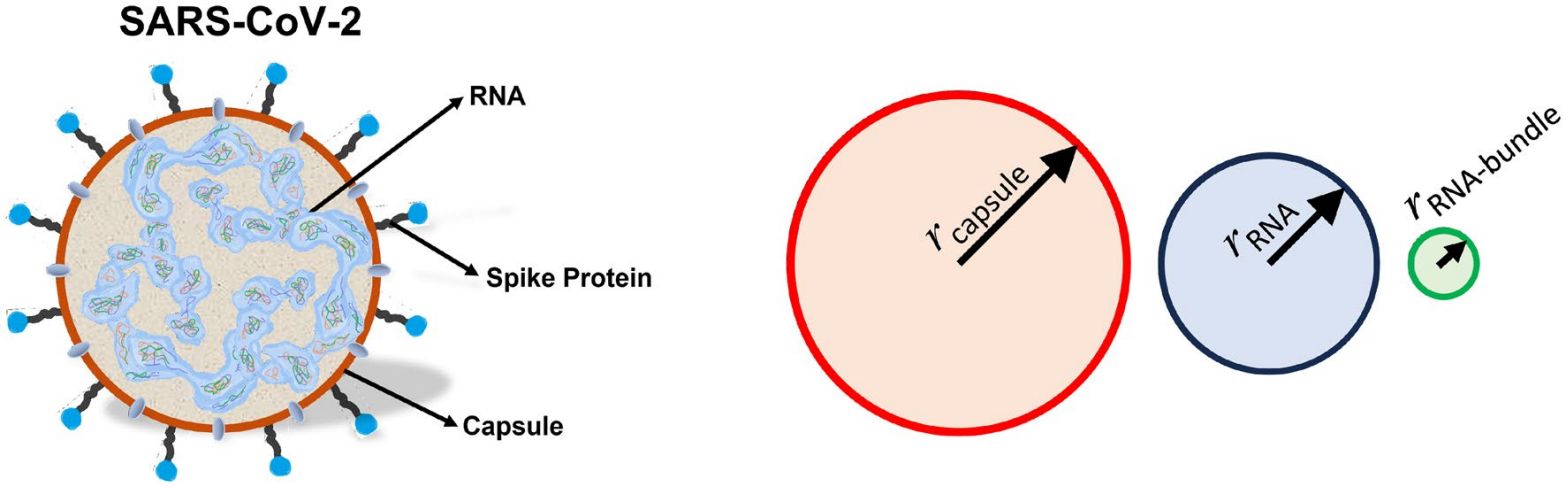
Left: structure of the SARS-Cov-2^19^. Right: comparative representation of the geometrical cross section of the capsule ($r_{\textrm{capsule}}$), the individual RNA bundle ($r_{\mathrm{RNA-bundle}}$), and the equivalent RNA bundle ($r_{\textrm{RNA}}$), as presented in Table 1.

Morphologically, the virus has two main parts: the capsule and the spikes (see Fig. 1). According to the literature^15^, the capsule can be considered as an ellipsoid with a semiaxis $a=32.4 \pm 5.9$ nm, $b=43.0 \pm 4.7$ nm, and $c=48.3 \pm 5.9$ nm. These values allow us to calculate the capsule’s volume and its uncertainty as

1 $$\begin{aligned} V_{\textrm{capsule}} = \frac{4}{3} \pi a b c \left[ 1 \pm \left( \frac{\Delta a}{a} + \frac{\Delta b}{b} +\frac{\Delta c}{c} \right) \right] , \end{aligned}$$

which results in $V_{\textrm{capsule}}= (2.82 \pm 1.17) \times 10^{-22}$ $\hbox {m}^3$. For simplicity, we consider a spherical capsule with equal volume to the ellipsoidal one. This equivalent spherical capsule has a radius $r_{\textrm{capsule}}= 40.7 \pm 5.6$ nm. The geometric circular cross section of the spherical capsule can be described as $\sigma _{\textrm{capsule}}= \pi r_{\textrm{capsule}}^2 = 5.20 \times 10^{-15}$ $\hbox {m}^2$. This description, using the geometric cross section, should be completed with the scattering and absorption cross sections. Unfortunately, the lack of reliable values for the optical constants of the different virus’ components and structures precluded an accurate analysis through computational electromagnetism. Other contributions^16,17^ also approximate the ellipsoid shape of the virus to a sphere with radius *r*, between 50 and 120 nm, where the spikes are also included. Cryo-electron tomography analysis^18^ shows how the ribonucleic proteins are organized in bundles with an almost spherical shape of $r_{\mathrm{RNA-bundle}}=8$ nm. These units can be arranged as a mixture of hexagonal and tetrahedral superstructures. The same study shows that the number of quasi-spherical clusters has a median value of $N_{\mathrm{RNA-bundle}}=33$. Using this arrangement, we have calculated the equivalent volume and geometric cross section taking a collection of 33 RNA bundles packed within the virus. The result is a value of $V_{\textrm{RNA}} = 0.75 \times 10^{-22}$ $\hbox {m}^3$, and $\sigma _{\textrm{RNA}} = 2.15 \times 10^{-15}$ $\hbox {m}^2$. We have summarized all geometric parameters in Table 1. A graphical representation of the capsule, individual RNA bundle, and equivalent RNA bundle cross sections is also presented in Fig. 1.

With respect to virus’ amount of matter, its mass is $M_{\textrm{virus}} \simeq 10^3$ MDa^20^, where Da is the unified atomic mass unit (i.e., 1 Dalton = 1Da = $1.66 \times 10^{-27}$ Kg). Inside the capsule, the most relevant structure for our model is the RNA chain. SARS-CoV-2 is a single-stranded RNA virus and its genome has a length of $L=29476$ bases of RNA^15,19–21^. We calculate the mass of the RNA chain using an on-line tool^22^, which provides an approximate mas of $M_{\textrm{RNA}}= 9.45$ MDa. The distribution of the nucleotides is A = 30 %, U = 32 %, C = 18%, and G = 20 %. This implies that the RNA chain represents a small portion $\rho _{\textrm{RNA}} = M_{\textrm{RNA}} / M_{\textrm{virus}}$, around 1%, of the total mass of the virus.

**Table 1:** Geometrical parameters of the SARS-CoV-2.

Parameter	Value (unit)
Capsule short semiaxis, *a*	$32.4 \pm 5.9$ [nm]
Capsule medium semiaxis, *b*	$43.0 \pm 4.7$ [nm]
Capsule long semiaxis, *c*	$48.3 \pm 5.9$ [nm]
Capsule equivalent spherical radius, $r_{\textrm{capsule}}$	$40.7 \pm 5.6$ [nm]
Capsule geometric cross section, $\sigma _{\textrm{capsule}}$	$(5.20 \pm 1.4) \times 10^{-15}$ [$\hbox {m}^{2}$]
Capsule volume, $V_{\textrm{capsule}}$	$(2.82 \pm 1.17) \times 10^{-22}$ [$\hbox {m}^3$]
Spike length, $L_{ \mathrm spikes}$	23 [nm]
Averaged number or spikes, $N_{\textrm{spikes}}$	26
RNA bundle radius, $r_{\mathrm{RNA-bundle}}$	8 [nm]
Median number of RNA bundles, $N_{\mathrm{RNA-bundle}}$	$33 \pm 2$
Equivalent RNA bundle radius, $r_{\textrm{RNA}}$	26.1 [nm]
Equivalent RNA bundle cross section, $\sigma _{\textrm{RNA}}$	$2.15 \times 10^{-15}$ [$\hbox {m}^2$]
Total volume of RNA bundles, $V_{\textrm{RNA}}$	$0.75 \times 10^{-22}$ [$\hbox {m}^3$]

### Estimation of the RNA chain inactivation locations

**Figure 2:**
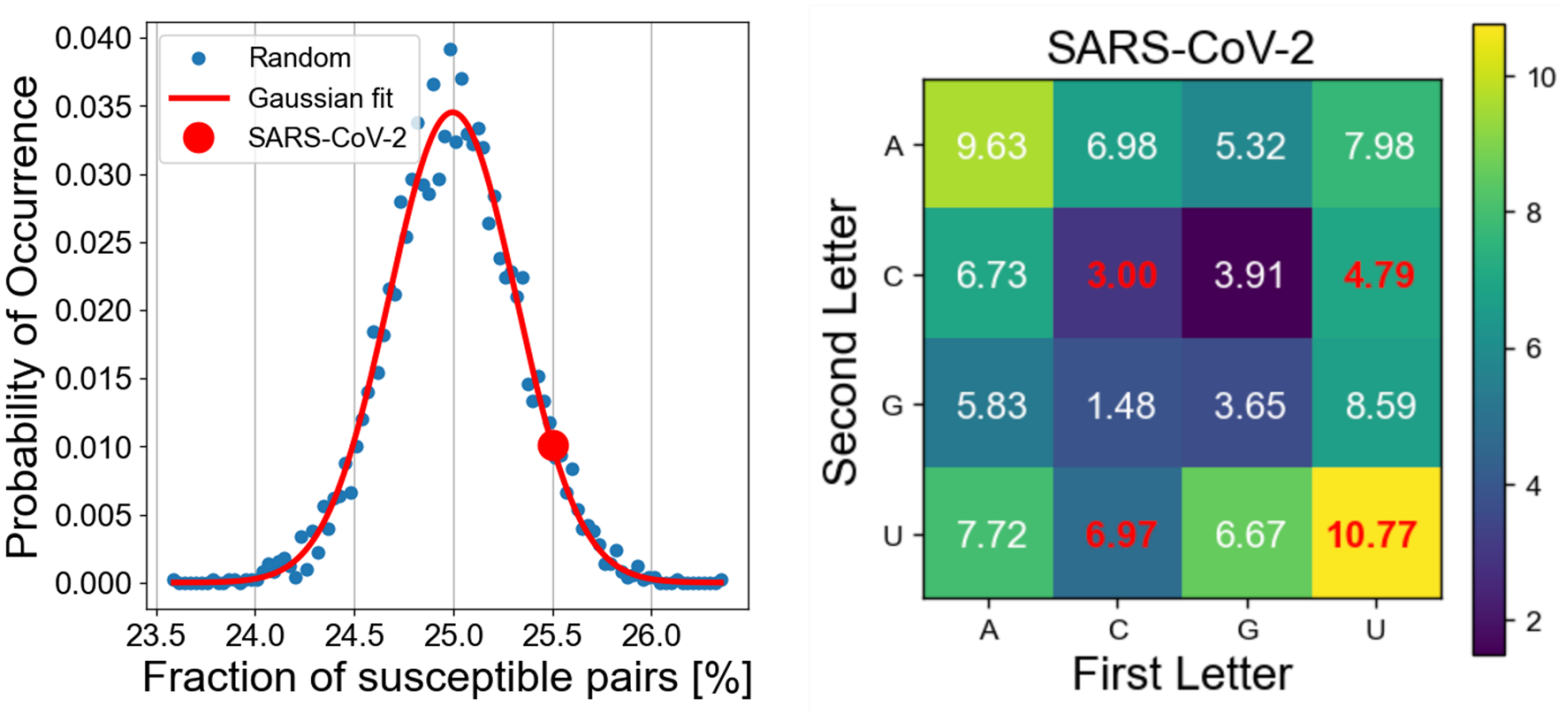
Left: normalized probability of occurrence as a function of the percentage of neighbors susceptible to UV light damage (blue dots). We have generated 5000 times a RNA chain of 30,000 bases randomly arranged. The red line represents the fitting of this distribution to a Gaussian curve with a mean equal to 25% and $\sigma =0.3$%. The large red dot corresponds with the case of the SARS-CoV-2 pathogen. Right: Distribution of the 16 combinations of neighbors for the SARS-CoV-2 RNA chain. The numbers in red correspond to the nucleotide pairs that are affected by the UV radiation and generate a disturbance in the base functionality: CC, CU, UC, and UU. These four nucleotide combinations represent 25.5% of the total number of neighbors.

Any RNA sequence is an ordered combination of nucleotides. RNA chains contain only four nucleotides: guanine (G), adenine (A), cytosine (C), and uracil (U). The presence of specific neighbors in the RNA chain is key for the virus’ photochemical inactivation^10,23^. These combinations are CC, CU, UC, and UU. These 4 pairs represent 25% of the 16 possible combinations of the 4 nucleotides. If we consider a random distribution of these four nucleotides within a chain with length *L*, the expected number of possible inactivation sites should be $\sim$25% of the total number of neighbors, that is $L-1$. To prove this, we generated 5000 realizations of a 30,000-nucleotide-long RNA sequence where we randomly selected the nucleotides along the chain. The plot on the left of Fig. 2 shows the probability distribution of occurrence of the four combinations of interest—those responsible for photochemical inactivation—within the computed RNA chain, as a function of the fraction of these pairs with respect to the $L-1$ total number of neighbors. We numerically fitted this distribution to a Gaussian curve with an average equal to 25 % and a width of 0.3 %.

The actual RNA chain of the SARS-CoV-2 has $L=29476$ bases. We downloaded the sequence from the National Library of Medicina webpage^21^. From these data, we have calculated that the RNA chain of the SARS-CoV-2 pathogen contains $M=7523$ locations of the nucleotide’s pairs susceptible to UV-C damage, which accounts for 25.5% of all neighboring pairs. The case of the SARS-CoV-2 is marked as a large red dot in the plot on the left of Fig. 2. The plot on the right of Fig. 2 shows the percentages of finding the 16 possible combination of adjacent bases in the analyzed SARS-CoV-2 chain where the susceptible of damage pairs are written in red.

## Inactivation model

The optical source’s capability to inactivate a given pathogen strongly depends on the pathogen itself. In the case of single-stranded RNA viruses, like SAR-CoV-2, the inactivation process can be modeled as an exponential decay of the number of surviving pathogen’s units^2^

2 $$\begin{aligned} N_s=N_0 \exp \left( - \frac{F}{F_i} \right) , \end{aligned}$$

where $N_0$ is the number of pathogens before irradiation and $N_s$ is the number of active pathogens after irradiation with a fluence *F*. The characteristic fluence $F_i$ is also related with the inactivation susceptibility, $k=1/F_i$, and with the D63 dose ($F_i = F_{\textrm{D63}})$, meaning that under this fluence 63% of the virus are inactivated. For a monochromatic source emitting at a fixed wavelength $\lambda$, this characteristic fluence can be transformed into a photon flux $\Phi _i= F_i/h\nu$, where the frequency is $\nu = c/\lambda$ (*h* is Planck’s constant and *c* is the speed of light). Both the fluence and the photon flux describe the optical radiation per unit area. Then, the number of photons, *n*, crossing an area $\sigma$ is given as $n = \Phi \sigma$. We can then transform Eq. (2) into

3 $$\begin{aligned} N_s=N_0 \exp \left( - \frac{n}{n_i} \right) , \end{aligned}$$

where $n_i$ is the number of photons corresponding to the characteristic fluence, $F_i$, for the area reached by *n* photons. Previous contributions have determined the characteristic fluence to be $F_i=4.7$ J/$\hbox {m}^2$^14^, which corresponds with a photon flux $\Phi _i=6.00 \times 10^{18}$ photons/$\hbox {m}^2$.

From Eq. (3) we can derive some simple relations when varying the number of photons and further understand the effect on the surviving pathogen’s units. Let us assume that we increase the number of photons from *n* to $n+ \Delta n$. If $\Delta n$ is positive, the number of surviving units will be reduced by $\Delta N$. Therefore, we can rewrite the previous relation as

4 $$\begin{aligned} N_s - \Delta N = N_0 \exp \left( - \frac{n+\Delta n}{n_i} \right) . \end{aligned}$$

After some simple algebra and assuming that $\Delta n \ll n_i, n$ (i.e., the variation of the number of photons is negligible compared with *n* and $n_i$), we obtain

5 $$\begin{aligned} \frac{\Delta N}{N_0} = \frac{\Delta n}{n_i} \exp \left( - \frac{n}{n_i} \right) . \end{aligned}$$

Equation (5) has two limiting cases: (i) $\Delta N=1$, where only one additional pathogen unit is inactivated; and (ii) $\Delta n=1$, where there is one additional photon crossing the pathogen. In both cases, it is possible to calculate the effect (the inactivation of pathogen’s units) or the cause (the variation of the number of photons) of those changes. This can be written as:

6 $$\begin{aligned} \Delta N= & {} N_0 \frac{1}{n_i} \exp \left( - \frac{n}{n_i} \right) , \hspace{0.67cm} \textrm{when } \hspace{0.1cm} \Delta n=1, \textrm{and } \end{aligned}$$

7 $$\begin{aligned} \Delta n= & {} \frac{n_i}{N_0} \exp \left( + \frac{n}{n_i} \right) , \hspace{1cm} \textrm{when } \hspace{0.1cm} \Delta N=1. \end{aligned}$$

Equation (7) considers only one additional inactivated virus and calculates the variation in number of photons, $\Delta n$, for a given value *n* and for an initial virus population $N_0$. A complete analysis should consider the situation of a single virus unit, which deserves a dedicated study and goes beyond the scope of this contribution. In the following, we show a model to better understand the case of a single virus exposed to UV-C radiation.

For electromagnetic radiation—or a flux of photons—to inactivate a pathogen, the propagating energy must interact with the RNA chain. Therefore, we must estimate the energy, or number of photons, impinging on the pathogen. As a first approximation, we can consider the number of photons crossing the area of the capsule of the virus: $\sigma _{\textrm{capsule}} = 5.20 \times 10^{-15}$ $\hbox {m}^2$. For the previously calculated D63 photon flux, $\Phi _i=6.00 \times 10^{18}$ photons/$\hbox {m}^2$, the resulting number of photons interacting with the capsule is $n_{i,\textrm{capsule}} = \Phi _i \sigma _{\textrm{capsule}} = 3.12 \times 10^{4}$. From here, using the equivalent cross section of the RNA bundles, $\sigma _{\textrm{RNA}}= 2.15 \times 10^{-15}$ $\hbox {m}^2$, we obtain the number of photons interacting with the RNA chain: $n_{i,\textrm{RNA}} = \Phi _i \sigma _{\textrm{RNA}} = 1.29 \times 10^{4}$. At this point, we assume that photons are absorbed when damaging a suitable locations, which total $M=7523$ pairs, as previously identified in “Estimation of the RNA chain inactivation locations” section. Therefore, the number of photons available for the *M* neighbors is $n_{i,\textrm{RNA}}$. This number of photons correspond with an inactivation level of 63% (for a fluence $F_i=F_{\textrm{D63}}$), and results in $\sim 1.71$ photons available for each susceptible location. However, we can still analyze the inactivation phenomena further by considering the probability distribution of the interaction of a very small number of photons reaching each one of the 7523 pairs. In our estimation, we considered that a maximum of 4 photons are available for each nucleotide pair of interest.

**Figure 3:**
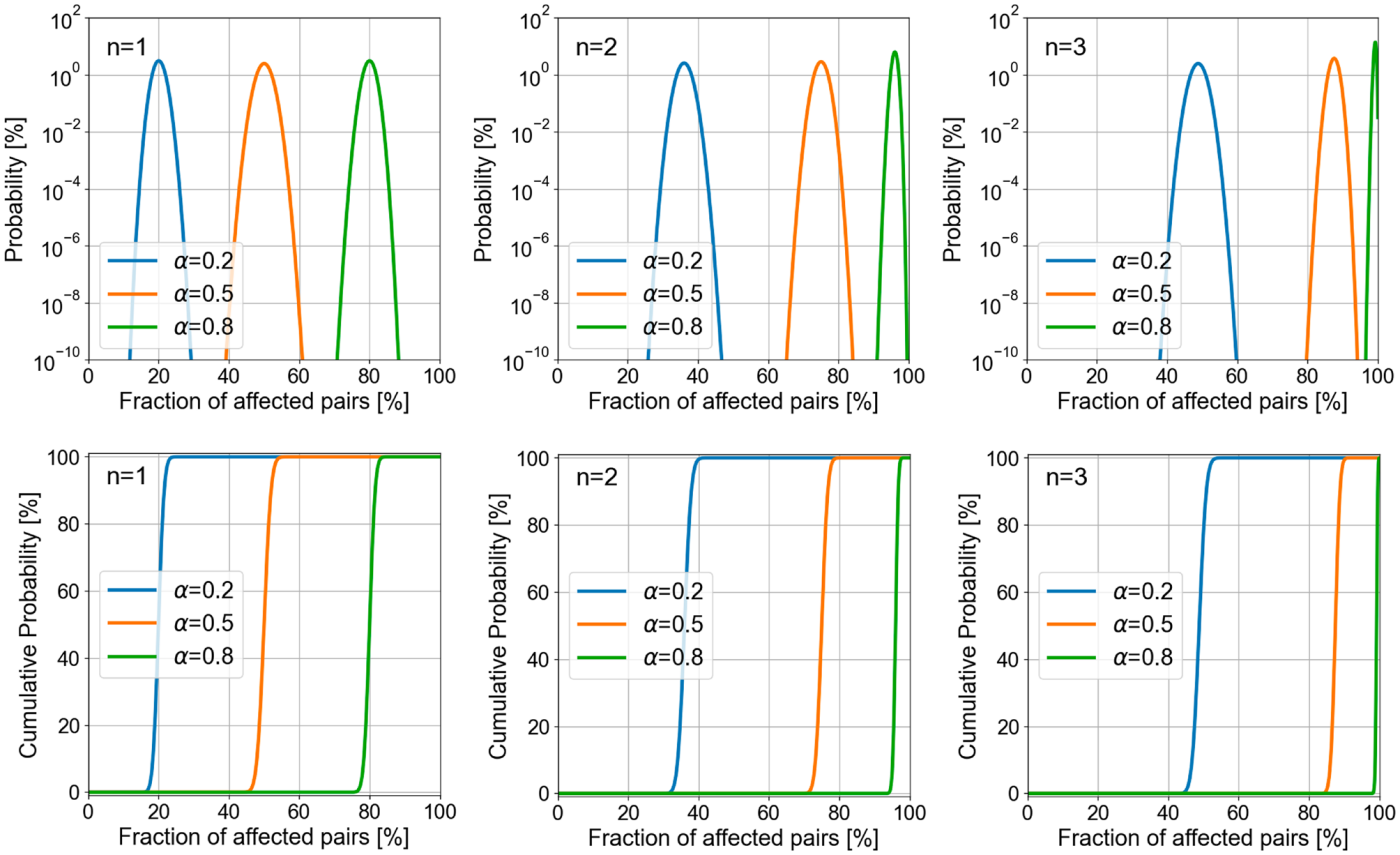
Probability (upper row in semilog scale), and cumulative probability (lower row) of having a given percentage of neighboring pairs damaged by radiation. The lines in color correspond to different values of $\alpha$ (0.2, 0.5 and 0.8 for blue, orange, and green, respectively), and each column represents a different number of interacting photons per pair, $n = 1, 2,$ and 3.

The probability of damaging a susceptible pair of neighbors by a single photon can be calculated in the same way as in a weighted coin tossing experiment. If we define $\alpha$ as the probability of damaging one pair of neighbors with one photon, the surviving probability would be $(1-\alpha )$. If this is repeated *n* times—*n* is the number of photons interacting with this pair—we can find that the probability of damaging the nucleotide pair is $(1-(1-\alpha )^n)$. If we have *M* pairs susceptible of damage, the probability of not affecting any pair would be $[ (1-\alpha )^n]^M$. So, the probability of damaging *k* pairs, *p*(*k*), would follow a binomial distribution:

8 $$\begin{aligned} p(k ) = \left( \begin{array}{cc} M \\ k \end{array} \right) \left[ 1 - (1-\alpha )^n \right] ^k \left[ (1- \alpha )^n \right] ^{M-k} . \end{aligned}$$

Due to computational limitations in the evaluation of large combinatorial numbers (see Eq. 8), we have limited the number of neighboring pairs to $M=1000$. In the upper row of Fig. 3, we show the probability of having a given ratio of these *M* pairs damaged by UV radiation. In these plots, we included several values of $\alpha$ ($\alpha = 0.2, 0.5$ and 0.8) and the number of photons interacting with each pair, $n=1, 2,$ and 3. The bottom row in Fig. 3 represents the cumulative probability for the same cases. Figure 3 shows that when $n = 1$, and $\alpha = 0.5$ (see orange line at the left upper plot), the maximum of the probability distribution occurs when 50% of the pairs suitable for damage are affected. For this case ($n=1$), the probability distribution shifts symmetrically around 50% when varying $\alpha$ away from 0.5. In fact, the ratio of affected pairs is equal to the probability of damaging a single pair, as should be expected when tossing a collection of *M* coins once. However, if the number of tossing increases (*n* increases), the probability distribution changes. Then, the maximum values of the probability distribution and the transition in the cumulative probability (see upper and bottom rows in Fig. 3) moves towards higher percentages of affected pairs when *n* increases.

**Figure 4:**
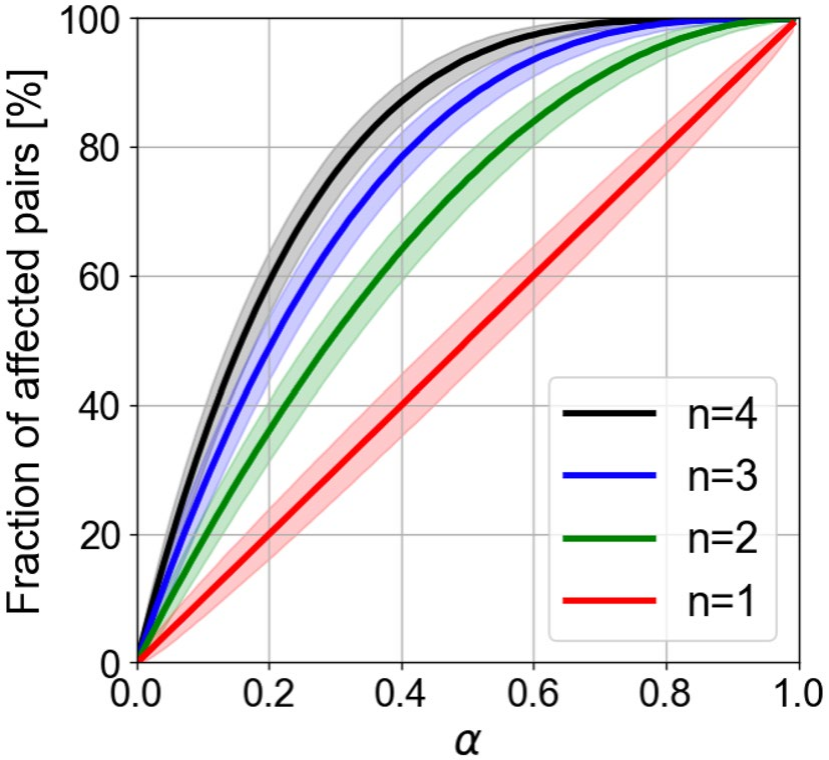
Percentage of affected pairs as a function of $\alpha$ for several values of the average number of photons, *n*, interacting with each pair. The solid lines represent the 50% cumulative probability, and the shaded region around them covers the portion between 0.1 and 99.9% in cumulative percentage (see bottom row in Fig. 3).

We observed that the cumulative probability shows a steep sigmoid shape (see bottom row in Fig. 3). This implies that it increases very rapidly from low to high percentages of affected pairs. Also, when the probability of damaging a single base’s pair, $\alpha$, increases, the sigmoidal transition moves towards a larger value of the percentage of affected pairs. This behavior is shown in Fig. 4 as a function of $\alpha$ for several values of the number of photons interacting with each pair susceptible of damage, *n*. The solid lines represent the 50% cumulative probability, and the shaded areas around them depict the region where the cumulative probability changes from 0.1 to 99.9% for several values of the averaged number of photons reaching each pair (from $n=1$ to $n=4$).

Unfortunately, to the best of our knowledge, we have no information of how many bases must be damaged for full pathogen inactivation. Therefore, we need to restrict our analysis to finding the combination of two parameters: $\alpha$, and *n*. At this point, we may safely assume that the virus is active when less than 0.1% of the pairs are affected (this corresponds to 7–8 pairs damaged in the RNA sequence), and inactive if 99.9 % of the nucleotide pairs are damaged (i.e., only 7–8 bases are unaffected by the UV-C radiation). Figure 4 shows narrow bands (represented as shaded areas) between these extreme percentages. Therefore, these shaded regions indicate when the RNA is damaged. As expected, the percentage of affected pairs within this transition increases when $\alpha$ and/or *n* increase. Our prediction is limited to the number of affected pairs damaged (by a given number of photons reaching each pair) as a function of the damage probability of a single pair, $\alpha$. Nevertheless, the actual probability of having an inactivated virus unit will depend on this distribution. As already shown, when having an average number of photons available for each neighboring pair $\sim 1.71$, a 63% inactivation probability is obtained. Therefore, if we consider 2 photons interacting with each base ($n=2$), the combination of the percentage of affected pairs and the value of $\alpha$ should lie within the shaded areas in Fig. 4. In the case of $n=2$ and $\alpha =0.5$, the range (in percentage) of affected bases lies between 71 and 79%; whereas for a value $\alpha =0.8$ (maintaining $n=2$) the range is from 94 to 98%. Regarding the number of photons per pair, *n*, a larger number of photons translates into more affected pairs.

In our calculations, we have checked that increasing the number of pairs, *M*, generates sharper transitions in the cumulative distributions. If we consider that the SARS-CoV-2 virus has 7523 nucleotide pairs subjected to damage by UV radiation, a larger value of *M* results in narrower shaded regions around the solid lines in Fig. 4.

## Conclusions

In this contribution we have considered the available information on the SARS-CoV-2 pathogen to analyze its photochemical interaction with UV-C photons, which enables pathogen inactivation. After evaluating the equivalent geometrical cross section of the virus’ inner structures where the RNA chain is stored, we proposed a calculation of the photon flux interacting with the virus instead of a classic electromagnetic calculation in terms of fluence. We considered the available genomic information of the virus to calculate the number of locations in the RNA chain that are susceptible to damage via UV-C light. We have compared the SARS-CoV-2 virus’ RNA sequence with randomly generated RNA sequences to understand better the occurrence of neighbors susceptible of UV damage. After evaluating the average number of photons for each selected locations, we proposed a binomial probability distribution to model the damage process of the affected pairs of nucleotides. This distribution shows a sharp maximum and a steep cumulative probability (in terms of the ratio of affected nucleotide’s neighbors), meaning that the transition between active and unactive RNA chains should occur within a narrow interval regarding the number of affected pairs of nucleotides. This calculation also revealed that the ratio of affected nucleotide bases to inactivate the virus strongly depends on the damage probability of a single pair and the available photons interacting with those pairs. We belileve these results may help to experimentally determine the number of affected bases necessary to inactivate the virus using UV-C light. Once this unknown is clear, and assuming that every susceptible-of-damage pairs are equivalent to inactivate the pathogen, a controlled irradiance experiment would provide enough information to estimate the damage probability, $\alpha$. Finally, although this analysis has focused on the SARS-CoV-2 virus, it can be applied to any other pathogen where the damage mechanism is related with the disruption of its nucleotide’s chain by UV-C light.

## Data Availability

The data used in this contribution has been obtained from scientific literature and publicly accessed sources as referenced in the text. The file containing the genome sequence can be downloaded from https://hdl.handle.net/20.500.14352/87787.
